# Genetic Association of the C-C Motif Chemokine Ligand 2 (CCL2) rs1024611 Polymorphism With Periodontitis

**DOI:** 10.7759/cureus.46438

**Published:** 2023-10-03

**Authors:** Hooriyah Laiq Ahmed Khan, Karthikeyan Murthykumar, Dhanraj Ganapathy

**Affiliations:** 1 Department of Prosthodontics, Saveetha Dental College and Hospitals, Saveetha Institute of Medical and Technical Sciences, Saveetha University, Chennai, IND; 2 Department of Periodontics, Saveetha Dental College and Hospitals, Saveetha Institute of Medical and Technical Sciences, Saveetha University, Chennai, IND

**Keywords:** dna markers, ccl-2, health care, polymorphism, chronic periodontitis

## Abstract

Introduction

To a large extent, a person's susceptibility to developing periodontitis is determined by their genetic makeup. Research has shown that chemokines generated during an immune response can harm the periodontal ligaments, gingiva, and alveolar bone. Various chemokine genes located on different chromosomes contribute to periodontitis, and one such gene is C-C motif chemokine ligand 2 (CCL2), associated with the rs1024611 polymorphism, which is part of a cytokine gene cluster on the q-arm of chromosome 17.

Objective

Our specific objective was to investigate whether CCL2 polymorphisms could influence the relative risk of developing periodontitis. Building on these findings, we aimed to compare the frequency of a specific single nucleotide polymorphism (SNP) in the CCL2 gene between individuals with and without periodontitis.

Materials and methods

Fifty participants were enrolled in the study after obtaining informed consent and ethical clearance. Clinical assessments, including probing pocket depth, clinical attachment loss, and bleeding on probing, were utilized to classify individuals into two groups: a control group (Group A, n=25) and a periodontitis group (Group B, n=25). DNA extraction from collected samples involved drawing 2 ml of venous blood from the antecubital fossa and transferring it into a sterile tube with a pinch of ethylene diamine tetraacetic acid (EDTA) to prevent clotting. DNA extraction was performed and polymorphisms of CCL2 were assessed through polymerase chain reaction (PCR) and restriction enzyme digestion.

Results

The periodontitis group consisted of 25 patients, with an average age of 39.0±0.22 years, who met the American Academy of Periodontology's 2018 criteria for at least stage II periodontitis. The control group comprised 25 individuals with an average age of 41.3±0.49 years. Regarding the CCL2 gene polymorphism (rs1024611), there was no substantial variation in genotype frequencies between the patients and controls (p = 0.695). An agarose gel electrophoretogram, along with a standard DNA ladder, demonstrated partial amplification of the CCL2 gene spanning the polymorphism site (rs1024611). Genotypes observed were as follows: homozygous AA - 333 bp; heterozygous AG - 333 + 250 + 73 bp; homozygous GG - 250 + 73 bp.

Conclusion

In conclusion, there is no significant association between the CCL2 gene polymorphism rs1024611 and susceptibility to periodontitis.

## Introduction

Periodontitis is characterized by chronic inflammation of the periodontal tissues, including the periodontal ligament, connective tissue, and alveolar bone. In addition to directly affecting host tissues, periodontitis acts as a persistent reservoir for inflammatory mediators and microbial products. Thus, chronic versions of the illness are related to a variety of systemic problems, such as cardiovascular disease, unfavorable pregnancy outcomes, rheumatoid arthritis, diabetes, and pulmonary diseases, in addition to their devastating local consequences [1]. It demonstrates a complex chain of causes [2]. In the early stages of periodontitis, the presence of Gram-negative anaerobic bacteria is crucial, although various environmental and genetic variables may influence the progression of the disease [2,3]. Periodontitis susceptibility is mostly determined by a person's genetic makeup [4,5]. Numerous investigations have looked for possible causes of periodontal disease susceptibility [6,7].

Periodontitis, an inflammatory illness of the gums, affects a large percentage of the global population. The bacteria that cause periodontitis secrete substances that stimulate the innate immune system, resulting in the production of proinflammatory chemokines that further advance the disease. In turn, this stimulates the acquired immune system, speeding up the development of periodontitis [8]. Chemokines secreted during an immunological response may wreak havoc on the periodontal ligaments, gingiva, and alveolar bone. Periodontitis is associated with many chemokine genes located on different chromosomes. C-C motif chemokine ligand 2 (CCL2) rs1024611 is located on the q-arm of chromosome 17, along with a number of other cytokine genes. The chemokine protein superfamily is a group of proteins released by cells that have important roles in immune regulation and inflammation. Based on the architecture of the mature peptide's N-terminal cysteine residues, the superfamily may be broken down into four subfamilies. As a member of the CC subfamily, this chemokine is distinguished by the presence of two consecutive cysteine residues [9]. CCL2, a chemokine, exhibits chemotactic action intended for basophils and monocytes. It has been linked to conditions such as psoriasis, rheumatoid arthritis, and atherosclerosis, all of which are characterized by monocytic infiltrates [10]. Signal transduction activities, including chemotaxis and the activation of inflammatory and bone cells, are triggered when specific receptors like CC chemokine receptor (CCR) 2 and CCR4 interpret chemokine signals. The severity of severe acute respiratory syndrome coronavirus 2 (SARS-CoV-2) infection is correlated with increased expression of the protein it encodes [11].

Chemotactic cytokines, or chemokines, play a key role in redirecting immune cells to an infected area of the body. In inflammatory diseases like periodontitis, chemokines are small polypeptides produced into the microenvironment that attract leukocytes and other immune mediators to the site of action [12]. New research points to a possible connection between periodontitis and oral cancer, in line with the hypothesis that chronic inflammation plays an important role in the development of both diseases [13]. Furthermore, several chemokines and their receptors have been linked to the initiation and progression of cancer by promoting cell proliferation, motility, angiogenesis, and metastasis, as shown in earlier research [14]. Previous studies have shown that in rheumatoid arthritis (RA), pro-inflammatory chemokines prevail over anti-inflammatory chemokines, leading to heightened inflammation in the synovial tissue. Given this knowledge, blocking chemokines either broadly or specifically may reduce synovitis [15]. Little research has examined the allele, genotype, or haplotype frequencies of CCL2 polymorphisms in people with periodontitis. Therefore, we anticipated that CCL2 polymorphisms could influence the relative risk for periodontitis. These findings from this research led us to examine the effects of a single nucleotide polymorphism (SNP) in the CCL2 gene in both the control and periodontitis groups.

## Materials and methods

**Figure 1:**
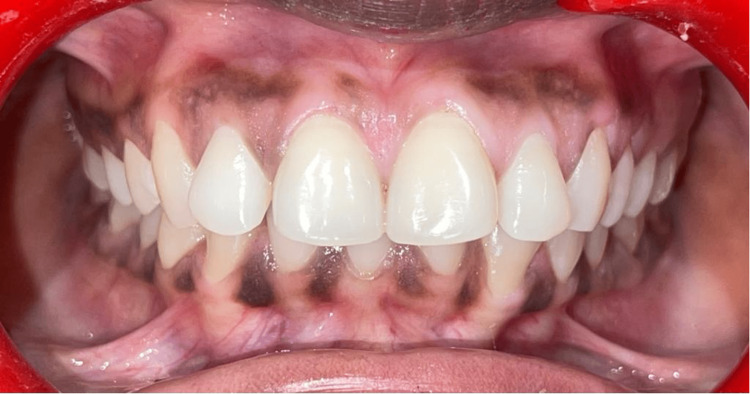
Representative image of human subjects with healthy periodontium

**Figure 2:**
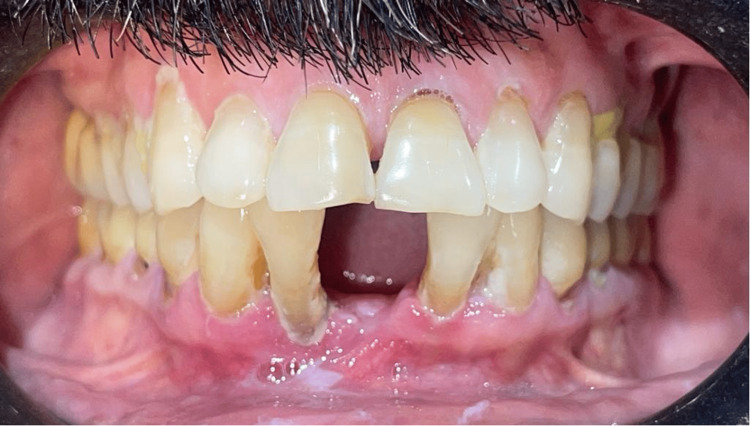
Representative image of human subjects with stage II periodontitis

Cross-sectional research was employed for this investigation of the South Indian population. Fifty individuals who reported to the Department of Periodontics participated in the study after obtaining informed consent and ethical clearance. Two groups were formed from the participants (Group A, n=25; Male=12, Female=13): healthy controls with no clinical manifestation of periodontal disease (Figure 1) and those with periodontitis (Group B, n=25; Male=14, Female=11) (Figure 2), as determined by clinical measurements of probing pocket depth, clinical attachment loss, and bleeding on probing. The periodontitis group consisted of 25 patients with a mean age of 39.0±0.22 years. Patients with periodontitis who were in stage II or above, according to the American Academy of Periodontology's 2018 criteria, were selected for the study. The 25 individuals in the control group had a mean age of 41.3±0.49 years.

Participants' dental treatment histories, family histories of periodontal diseases, smoking habits, and general health concerns were recorded. The participants in this research were generally healthy except for the presence of periodontitis. Individuals who met the following criteria were excluded from the study: current smokers, women who were breastfeeding at the time of enrollment, people with weakened immune systems, and those who had undergone periodontal treatment within the previous six months.

PCR analysis

The antecubital fossa was pricked, and 2 ml of venous blood was drawn and poured into a sterile tube with a dab of ethylene diamine tetraacetic acid (EDTA) to prevent clot formation. DNA was isolated and PCR amplification and restriction digestion were used to evaluate CCL2 rs1024611 polymorphisms. DNA spanning the gene was amplified using the following forward and reverse primers: 5'-GGGAGGGCATCTTTTCTTGA-3' and 5'-AAAGTGACTTGGCCTTTGCAT-3', respectively. The primers were designed using PrimerBLAST. DNA was amplified in a 20 ul volume using 10 ng of genomic DNA, 5 pmol/ul of forward and reverse primers, and a PCR master mix. The temperatures and times for each step of the cycling process were as follows: initial denaturation for 5 minutes at 94°C, followed by denaturation for 35 seconds at 94°C, annealing for 35 seconds at 60°C, and finally extension for 5 minutes at 72°C. A 1% agarose gel was used to examine 5 μl of PCR product. Restriction enzyme pre-degree celsius curing from New England Biolabs (Ipswich, MA, USA) was used to digest 15 μl of PCR product. There was a two-hour period of digestion at 37°C. The results of the digestion were observed on an agarose gel with a concentration of 2%.

Analytical statistics

The Statistical Package for the Social Sciences, version 23.0 for Windows, was used for all statistical analyses (IBM Corp., Armonk, NY, USA). The power estimation was obtained utilising the help of a statistician and statistical power of 80% was established for the present study. Chi-square analysis was applied to examine the frequency of genotypes and alleles in the periodontitis and control groups. We used odds ratios (OR) with 95% confidence intervals to determine the risk associated with different alleles and genotypes. In all analyses, significance was set at the P < 0.05 level.

## Results

The genetic association of CCL2 (also known as MCP-1) polymorphism with periodontitis has been a subject of interest in periodontal research. CCL2 is a chemokine that plays a key role in the recruitment and activation of monocytes and macrophages, which are involved in the inflammatory response. Polymorphisms in the CCL2 gene can influence its expression and function, potentially impacting the development and progression of periodontitis. Table 1 presents allele and genotype frequencies of the CCL2 gene polymorphism (rs1024611) in both patients and controls. A one-way chi-square test was used to evaluate deviations from Hardy-Weinberg equilibrium (HWE). The analysis revealed minimal variation in genotype frequencies between patients and controls (χ2df, P = 0.695), indicating that the genetic distribution in the study population closely adheres to HWE expectations.

**Table 1:** CCL2 gene polymorphism (rs1024611) allele and genotype frequencies in patients and controls *One-way chi-square to measure deviation from Hardy-Weinberg equilibrium (HWE). The genotype frequency of patients and controls do not vary substantially χ2df (P = 0.695). A: Adenine, G: Guanine.

Groups	AA	AG	GG	A	G	HWE (p value)*
Case (N=25)	12	9	4	0.66	0.34	0.322
Control (N=25)	15	7	3	0.74	0.26	0.173

Nextly, we depicted the polymorphism in the CCL2 gene and its distribution across the chosen control and case samples (Table 2). 

**Table 2:** Polymorphism in the CCL2 gene (rs1024611) and its distribution across cases and controls. A: Adenine, G: Guanine.

Dominant				
Genotypes	Case	Control	Unadjusted OR (95% CI)	P value
AA	12	15	0.622 (0.179-2.162)	0.455
AG+GG	9	7		
Recessive				
AG+AA	21	22	0.715 (0.142-3.588)	0.684
GG	4	3		
Allele				
A	33	37	0.682 (0.288-1.613)	0.383
G	17	13		

**Figure 3:**
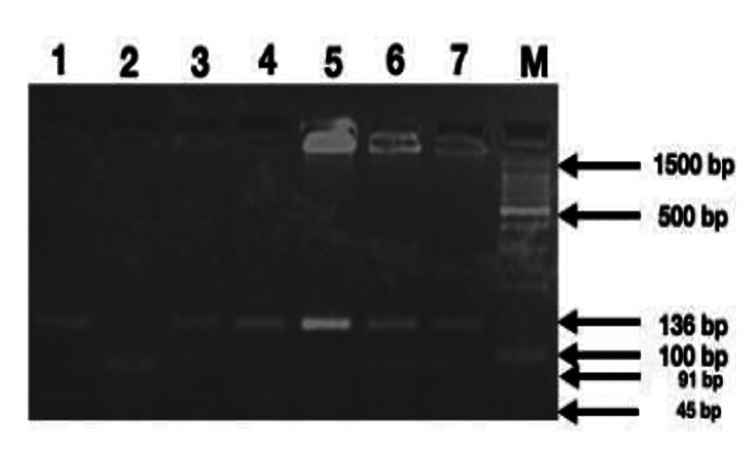
Agarose gel electrophoretogram showing PvuII digested amplicon of CCL2 spanning the rs1024611 site (Homozygous AA - 333 bp; Heterozygous AG - 333 + 250 + 73 bp; Homozygous GG- 250 + 73 bp) (Lane M = 100 bp DNA marker; 1-7 indicates PCR-amplified samples collected from patients) A: Adenine, G: Guanine, PCR: polymerase chain reaction, bp: base pairs

**Figure 4:**
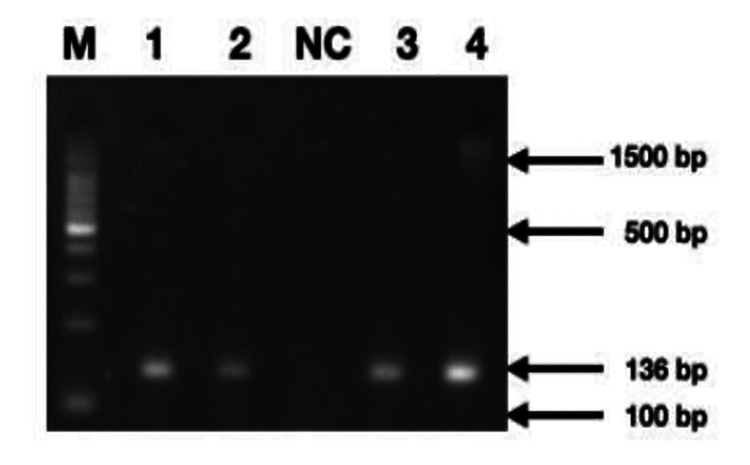
Agarose gel electrophoretogram showing partial amplification of CCL2 gene (bands between 136 and 100 bp) spanning polymorphic site (rs1024611) run along with standard DNA ladder (Lane M = 100 bp DNA marker; NC: Negative control; 1-4: Test samples). bp: base pairs

**Figure 5:**
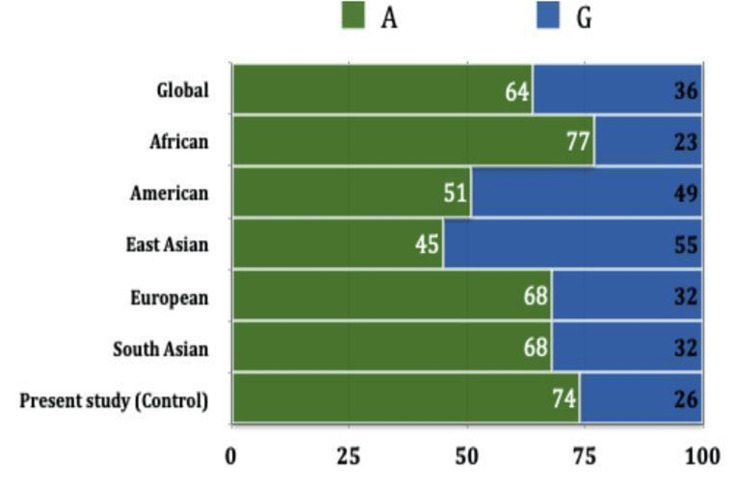
The graph depicts the allele frequency of CCL2 gene polymorphism (rs1024611) in different populations (Data acquired from Ensembl database, 2022). A: Adenine, G: Guanine

We conducted agarose gel electrophoresis of samples digested with the PvuII enzyme, and we successfully identified the amplicons of CCL2 in these samples (Figure 3). Given the established association of CCL2 polymorphism with periodontal disease, we further confirmed the frequency of CCL2 polymorphism in our sample using PCR (Figure 4). Additionally, we extracted allele frequency data for CCL2 polymorphism from the Ensembl database and represented it in Figure 5 for comparison with different populations.

## Discussion

Environmental factors play a significant role in initiating periodontitis, an inflammatory condition resulting from interactions between the host and oral microorganisms. Various environmental variables, such as smoking, infections, socioeconomic status, systemic factors like diabetes and other inflammatory disorders, stress, obesity, and hereditary factors, all seem to contribute to the development of periodontitis. Moreover, the rate at which the disease progresses can vary from person to person, with aggressive and rapidly progressing forms of periodontitis believed to be influenced by genetic susceptibility [16].

Genetic polymorphism accounts for phenotypic diversity within a species, defined as having more than one variant of a gene in a population at a frequency of 1% or higher. It's important to note that polymorphism distribution can vary widely among different groups, making it challenging to extrapolate findings from one population to another. Studies have explored histocompatibility antigen polymorphisms, IgG class antibody receptor polymorphisms, CD14 molecule polymorphisms, toll-like receptor polymorphisms, vitamin D receptor polymorphisms, and other cellular receptor polymorphisms in relation to chronic and aggressive periodontitis. Additionally, various polymorphisms in genes encoding metalloproteinases and other enzymes have been analyzed. However, most attention has been given to variations in genes encoding pro- and anti-inflammatory cytokines [17]. Multiple components of the inflammatory and immunological response are known to play roles in periodontitis development. Identifying genes contributing to the etiology of periodontitis could improve risk assessment for both aggressive and chronic forms of the disease. SNPs are a type of genetic variation that can impact disease in complex ways by interacting with other genetic variants and environmental factors, influencing disease susceptibility and progression. Several studies have linked SNPs to periodontitis risk [17].

Our research revealed no significant variation in the frequency of CCL2 polymorphism genotypes between the periodontitis and control groups. There were no statistically significant differences in the frequencies of either homozygous or heterozygous mutant genotypes. Neither the AG nor the AA genotypes were more prevalent in the periodontitis group compared to the healthy controls. The frequencies of the A allele and the G allele did not differ between the periodontitis and healthy control groups. These findings suggest that CCL2 may not serve as a biomarker for periodontitis. In the context of inflammation, CCL2/CCR2 signaling is best known for its role in controlling macrophage recruitment and polarization, influencing macrophage chemotaxis and adhesion through activation of integrins and p38MAPK signaling pathways. Consequently, the pathological degradation of the extracellular matrix associated with periodontal disease is related to CCL2 production and activation. CC chemokine monocyte chemoattractant protein (CCL2) plays a role in inflammation by modulating fibroblast and endothelial cell phenotypes [18]. These hypotheses drove the current investigation into potential links between CCL2 gene polymorphism and periodontitis.

Unlike the CCR532 polymorphism, which may or may not be involved in periodontitis, Shih et al. found that the CCL5-403 G substitution by A may play a role in periodontitis [19]. Alleles A and G of the CCL5-403 polymorphism were significantly associated with different subtypes of periodontitis. The study revealed that allele A, rather than allele G, was more prevalent in CCL5-403 in generalized aggressive periodontitis (GAgP) patients, with a rate 3.7 times higher than in chronic periodontitis (CP) patients and 2.0 times higher than in chronic gingivitis (CG) patients. GAgP patients were 3.1 times more likely to have an AG genotype than CG patients. Additionally, GAgP patients were five and 19 times more likely to have the AG and AA genotypes, respectively, compared to CP patients with the GG genotype. These variations might be explained by the larger sample size and the ethnically diverse participant group. Qi et al. also found associations between the rs2230054 and rs1126580 polymorphisms in the CXCR2 gene and peri-implantitis susceptibility in the Chinese Han population [12]. The risk factors for severe peri-implantitis included the CT genotype of rs2230054 and the AG genotype and G allele of rs1126580. Differences between the present research and previous studies may be attributed to sample size and participant ethnic diversity.

Limitations

This study acknowledged potential variations in genetic polymorphisms among different ethnic groups. It's crucial to recognize that both the sample size and the ethnic composition of the study population can significantly impact the results. To draw more generalizable conclusions, larger and more diverse samples are required. Genetic susceptibility to periodontitis is likely influenced by multiple genes, each with various polymorphisms. While this study focused on CCL2, it represents only one aspect of the larger genetic picture. Future research should explore the interactions between different genetic variants and their combined effects on disease risk. Additionally, a comprehensive analysis should integrate genetic and environmental variables to gain a better understanding of disease susceptibility. Longitudinal studies would be valuable for understanding how genetic factors interact with disease progression. Furthermore, it's important to note that genetic associations can vary between different populations. Therefore, findings from one study may not be directly applicable to other ethnic or geographic groups.

## Conclusions

In the future, multicenter investigations involving diverse cultural backgrounds will be essential to provide a more comprehensive understanding of CCL2 gene polymorphism. The current research had the limitation of including participants from a single ethnic background, and expanding the scope is crucial to ensure broader applicability. One of the strengths of the present study lies in its meticulous screening of potential confounding variables, such as smoking and systemic diseases, within the sample. To gain a thorough understanding of the etiopathogenesis of periodontitis, further research should delve deeper into the intricate interplay of genetic, microbial, and environmental factors. The findings from this research suggest that the rs1024611 polymorphism in the CCL2 gene is not significantly associated with an increased risk of developing periodontitis. However, to confirm these results, additional studies are warranted. These future investigations should explore the functional mechanisms of the SNP and involve diverse ethnic communities to ensure the robustness and generalizability of the findings.
